# Users' and researchers' construction of equity in research collaboration

**DOI:** 10.1111/hex.13026

**Published:** 2020-01-20

**Authors:** Susanne Stuhlfauth, Ingrid Ruud Knutsen, Christina Foss

**Affiliations:** ^1^ Department of Nursing Science Faculty of Medicine University of Oslo Oslo Norway; ^2^ Department of Nursing and Health Promotion Faculty of Health Science Oslo Metropolitan University Kjeller Norway

**Keywords:** focus group, positioning theory, qualitative research, social construction, user involvement in research

## Abstract

**Background:**

Equity is described as an ideal in user involvement in research and is mentioned in the health service literature and in several guidelines. However, equity is described as being difficult to obtain and the concept is rarely clarified or concretized. Equity can be socially constructed.

**Objective:**

This study explored users' and researchers' constructions of equity in research processes.

**Design and Method:**

The study had a qualitative research design. Constructions of equity were analysed through the lens of positioning theory. Two focus group interviews consisting of both users and researchers were conducted.

**Findings:**

The thirteen users and four researchers considered ‘equity’ as an important part of user involvement in research. Storylines about norms, responsibility, language, knowledge and usefulness evolved in the discussions. These storylines elucidated unequal access to rights and duties.

**Discussion and conclusion:**

Users and researchers constructed equity in user involvement differently, but the difference was masked by an apparent agreement. Users and researchers drew on different storylines. The researchers emphasized the scientific discourse and although users acknowledged this discourse, they attempted to oppose this dominant discourse by drawing on a lay discourse. The identified constructions and negotiations of equity may contribute in new understandings of an equal collaboration in user involvement in research.

## 1 INTRODUCTION

User involvement is progressively becoming a routine element of health service research and is increasingly a common requirement for research funders.^1^, ^2^, ^3^ Although the importance of involving patients and the public in health‐care research is recognized, reviews of the literature find that users' roles are more often consultative than collaborative.^4^, ^5^, ^6^ Moral,^7^ political,^8^ ethical^9^ and democratic^10^ values are described as important for user involvement in research, and values related to respect, partnership and equity are often raised in the literature.^11^ Of these values, equity is often described as central in the collaboration process within current policies and stands out in the literature as a major concern.^12^, ^13^, ^14^, ^15^, ^16^, ^17^, ^18^ Health service literature indicates that users and researchers both describe equity as vital without elaborating on what it means to be equal partners in the context of user involvement in research.^11^ Equity has been described as a ‘blurred concept’^19^ and Gradinger et al^11^ stressed the need to further explore values such as equity.

To examine how equity is perceived within user involvement in research, it is required that we understand how power, rights and duties are played out in the interaction between users and researchers. The description of equity as an important concept is understandable as equity is strongly related to power issues.^20^, ^21^ Power can be seen as an overall factor in user involvement, as a stated aim of user participation is to strengthen democratic rights and to improve health‐care services.^22^ Although equity has been portrayed as the ideal for research collaboration, we have not been able to identify any research that explicitly investigated what users or researchers mean when they talk about equity. Furthermore, there is little description of any potential differences in stakeholders' views, which makes it difficult to know whether users and researchers have differing understandings of equity that might cause misunderstandings.

The vagueness of the concept of equity also became clear to us during a previous qualitative study, when we interviewed researchers and users on their experiences of participation in research projects.^19^ Our experiences from this focus group study were that the understandings of values related to user participation appeared to be continuously shifting, shaped and re‐shaped as a result of the on‐going processes. This made us see equity as a fluid concept that is socially and contextually constructed. With this understanding as a backdrop, the current study provides an in‐depth examination of equity in the research process by exploring users' and researchers' constructions.

### 1.1 Literature review

Terms such as ‘equal’, ‘equality’ and ‘equity’ are commonly and sometimes interchangeably used in health service literature^23^ to discuss the relationship between researchers and users.^11^, ^24^, ^25^ This literature often portrays the user as a partner in a collaborative research process, which implies the ‘sharing of power’ as essential for achieving successful user involvement.^26^, ^27^, ^28^ The designation ‘co‐production’ has been used in recent health service literature as a key factor in discussions of equity and partnership^13^, ^18^ INVOLVE defines co‐production as ‘an approach in which researchers, practitioners and the public work together, sharing power and responsibility from the start to the end of the project, including the generation of knowledge.’^14^ In this definition, power is depicted as the ‘holder’ of equity by constructing user involvement as a question of sharing power and influence. Several guidelines focus on issues related to partnership and power‐sharing in user involvement in research in an attempt to address the challenges which have been identified within user involvement in research.^2^ However, even though co‐production is portrayed as desirable, it is also described as utopian.^13^

The literature describes factors that promote and hinder an ‘equal collaboration’. The former includes coaching of researchers;^29^ provision of structures;^28^, ^30^ dynamics of learning processes;^28^ and an atmosphere of trust, respect and transparency.^9^, ^31^ Limited time,^11^, ^28^ lack of funding,^8^ mismatched expectations,^28^ negative attitudes^6^, ^9^ and differences in status^32^ are described as negatively influencing an ‘equal collaboration’. While some studies underline the need to share power,^5^, ^33^ others pay attention to the ways power is played out by using different theoretical approaches.^19^, ^21^, ^32^, ^34^ Different studies aiming to explore power in research collaboration tend to draw the conclusion that power hierarchies still exist.^19^, ^21^, ^32^, ^34^

## 2 THEORETICAL AND METHODOLOGICAL FRAMEWORK

The way equity is described (and practised) may be seen as reflecting current perceptions of user involvement in research. Discourses of user involvement are both shaped by and shape what becomes a common sense understanding of user involvement. Hence, in the process of interaction, users and researchers will use language to act, behave and speak that is influenced and guided by the social norms belonging to the existing discourses of user involvement. The ambition to study equity in user involvement draws our attention towards ways of understanding ‘language in use’*,* a phrasing that underlines a ‘saying‐doing’ combination.^20^ This combination underlines that language is shaped—and shapes practices—through how it is used and influenced by social norms and values.

**Figure 1:**
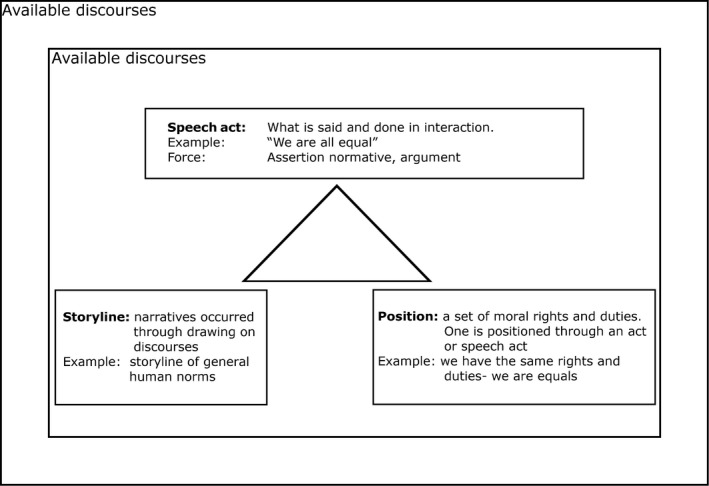
Example of the analysing process: The users' positioning act

We consider positioning theory an appropriate framework for this study as it has a strong emphasis on power and (in)equality^20^ by focusing on how rights and duties are accessible to users and researchers. Individuals, such as researchers and users, have rights and duties that are connected to their positions. There are three mutually determined elements central to positioning theory: speech act, position and storyline.^20^, ^35^ Position refers to a momentary cluster of rights and duties to act or speak reflecting an individual's sense of moral rights and duties,^20^, ^36^ and differences in the rights and duties reflect a differential distribution of power. For instance, if researchers, in contrast to users, are ascribed explicit responsibility for conducting the research process, this gives the researchers more powerful positions. Positions are expressed through speech acts and are brought forward within a storyline (Figure 1). In line with Kayi‐Aydar^20^, we see storylines as different narratives occurring around a topic (such as equity). Storylines may draw on several different discourses and play a key role in how subjects position themselves.^37^

### 2.1 Aim

This article aims to explore and describe how equity is constructed through the emerging storylines that users and researchers draw upon.

## 3 DESIGN AND METHOD

We employed a qualitative, explorative and descriptive design. Positioning theory focuses on interaction;^38^ hence, we considered focus group discussions to be an appropriate method for data collection. Opinions stated in a group, in our case about equity, are not seen as previously formed, static things that the informants have brought to the group but as constructed in the group setting.^39^

### 3.1 Recruitment

This study springs from experiences from focus group interviews in an earlier study.^19^ We invited the same groups back through a written letter, stating that the aim was to increase our understanding of equity. The letter emphasized that we would be searching for nuances, variations and divergences in their perceptions of equity.

### 3.2 The focus group method

Heterogeneous groups are recommended by several researchers to stimulate discussions and tease out potential differences in views and attitudes and elicit multiple nuanced meanings.^40^, ^41^, ^42^

Thirteen users and four researchers, all experienced in user involvement in research, accepted the mailed invitation (Table 1). Two focus groups were organized as smaller groups allow for contributions from each informant and ensured access to a variety of opinions.^40^, ^41^ Instead of expanding the sample, we decided to aim at obtaining rich data by stimulating the discussions to unfold through pursuing statements in‐depth and seeking different points of view. We considered that the informants had been acquainted with each other in a previous focus group study an advantage that might contribute to open and active discussion.

**Table 1:** Group participants

Focus Group 1	
User representative	User organization
Male	Prostate Cancer Organization
Female	Personal Injury Organization
Male	User Organization in a hospital
Male	Teeth and Health Organization
Female	Breast Cancer Organization
Female	Diabetes Organization
Female	Haematological Cancer Organization

Researcher	Research field
Female	Health services and Huntington disease (Psychologist)
Female	Rheumatological rehabilitation (Ergotherapist)

Focus Group 2	
User representative	User organization
Female	Lymphoma Organization
Female	Diabetes Organization
Male	Prostate Cancer Organization
Female	Gynaecological Cancer Organization
Female	Cancer Society
Female	Personal Injury Organization

Researcher	Research field
Female	Health care and environment (Sociologist)
Female	Nursing research (Nurse)

The first author led the focus group and the third author, an experienced researcher, observed and asked follow‐up questions. We used a semi‐structured interview guide to pursue equity among others through questions about status, respect, knowledge and partnership, all the time, following‐up the informants' statements. We encouraged participation from all the informants, and the moderators were active in the discussion to help clarify similarities and differences in expressed opinions. We invited the informants to describe their perceptions in detail, and they were encouraged to comment on the other informants' contributions to help clarify differences and nuances in their opinions.

### 3.3 Data analysis

We based our analytical approach on an understanding that the participants' thoughts on equity in user involvement were constructed through existing interacting norms and ideas that were constructed through different discourses.^43^ Individuals recognize themselves as either user or researcher and construct themselves and others according to existing discourses on user participation. Through the analysis of these interactions, we had access to the realities that are constructed through speech acts; how equity in user participation is not only expressed but also ‘done’*.* The analysis focused on a micro‐level approach that emphasized the local and contextual interaction in a focus group. However, to understand the positions of equity within particular storylines, we also had to focus on how the storylines point to power, privilege and status. Furthermore, we had to focus on what these positions do, for instance, marginalize or heed certain beliefs or practices.

In the analysis, the first author listened to the audio recordings several times and transcribed them verbatim. All the authors took notes and identified speech acts related to equity. We looked at the details of every interaction and the force of the speech acts through which positions were ascribed and assumed. Focusing on positions and the linked distribution of rights and duties helped us focus on the dynamic power aspect in the encounter and how this affected equity between users and researchers. By focusing on the narratives that unfolded through the discussion, we proposed tentative storylines. For instance, one such storyline was the users' and researchers' right and duty to be treated as equals (Figure 1). We moved back and forth between the layers of analysis, transcripts, research aims and the analytical concepts of positioning theory. Whiteboards and sketch pads were used to illustrate the storylines, positions and speech acts as they contributed to more flexibility compared with using software applications. All the authors were involved throughout the process, notes were taken during the meetings and we discussed various interpretations until we reached an agreement.

## 4 FINDINGS

The two groups consisted of six and seven users, respectively, and there were two researchers in each group. The participants stated that they considered equity as a highly relevant and interesting topic, a view that was confirmed through their engagement.

We identified four overall storylines that arose during the informants' discussions of equity: storylines of norms, responsibilities, knowledge and usefulness. The storylines elucidated experiences of unequal access to rights and duties, which also implied unequal power to promote certain storylines.

### 4.1 Storylines of norms

*As humans, we are equal* is a quotation that reflects similar other utterances from the users. By drawing on the ethical norm that all humans are equal, the users positioned themselves as having the right to equity by virtue of being human. Drawing on this human norm can be considered a means of reducing the differences related to position that occur in this context. The storyline was further elaborated by another user:

Equity means respecting you as a human as you do to me: that we are equal in the way we come together and talk together and listen to each other. I respect what you say because you do the same to me, and that is equity. (User)

Heeding the moral duty to be treated and to treat others ‘as humans’ might be seen as a way of creating distance from the research context by focusing on human norms. The researchers, however, were clearly more focused on the context in their emphasis on duties and, thus, responsibilities.

One researcher stated:

I don't mean that you, as a researcher, should not show compassion and care. But at the same time, if user involvement actually muddles for what is going to be explored in the project… (Researcher)

The phrase if ‘user involvement actually muddles’ might contradict the users' utterance that everyone respects each other and exemplifies different views between users and researchers.

The term ‘respect’ was frequently used in descriptions of equity from both parties, indicating that respect is vital in the construction of equity. One of the users stated:

Equity, to me, means that you are respected and taken into the conversation on equal terms with the others.' Another user defined equity as having the respect to talk things through.

Being respected is related to being part of discussions (on equal terms with researchers) instead of being met with ‘polite silence’. Respect was also seen as implying integrity, as illustrated by the following utterance:

When you talk about equity, you talk about equity as humans, and that is all about respecting one another's integrity…. Equity is all about acknowledgement and respect for the organization and the diagnoses that I represent, so it is on several levels. (User)

The word ‘integrity’ connotes honour, honesty, strong moral principles and an individual's right to express his/her own thoughts and opinions.

The extent to which social relations affect equity was also a topic. The informants discussed whether acting as equals implied friendship, collegiality or other types of relationships. One discussion initiated by the researchers was related to their clinical backgrounds, which were defined by a ‘patient‐professional’ relationship with distinct roles for each party.

In the following statement, one researcher actually expressed surprise at the shift in her position as a friend and as a colleague:

Some of the users have actually become friends, just like colleagues, but not all of course. And I have been interacting privately with them.

The use of the qualifiers ‘actually’ and ‘just like’ may suggest that the researcher did not consider the users to be colleagues yet. This implies that changing or leaving a ‘traditional’ position, where the researcher is in a powerful position and not an equal partner, might be challenging. Creating new positions was also challenging for the users. One user stated:

When we have meetings before we start working, we start with a pizza to be a bit social. That made me feel like a friend.

Thus, social events seemed to be an opportunity for the users to be acknowledged as the researchers' equals. Again, reducing power differences related to formal position and drawing on a storyline resembling everyday practices.

The above is an example of a negotiation between the ‘general human norms’ and the ‘research context‐specific norms’. The users' storylines involved respect, integrity and social relationships. The researchers, however, positioned themselves in a storyline in which general norms were not the primary focus, even though they did not directly oppose them. Most of the statements around norms were from the users, as they were the most active informants in this discussion.

### 4.2 Storylines of responsibility

Several storylines of responsibility emerged in the interaction between users and researchers. The users described a dependency on the researchers that seemed to be related to the researchers' responsibilities in the research project.

One user stated:

With regards to user involvement in research, one has a different responsibility in that process. Researchers are responsible for driving this process and likely have an idea of how things should be.

This utterance was supported by a researcher:

The project manager has the overarching responsibility and, in a way, the responsibility to keep the participants within the scope of the project.

In the foregoing excerpt, the researchers are ascribed responsibility by the users; however, they also assume positions of responsibility with regard to both the users and the project. The researcher's reference ‘keep the participants within the scope of the project’ refers to the project manager's responsibility and right to determine the scope. Thus, the researcher constructs responsibility as being connected to power and assumes a position with more power.

The users did not appear to reject the researchers' responsibility; on the contrary, they saw the researchers as facilitators who ensured that everyone felt comfortable.

One user stated:

The researchers need to take responsibility, and they need to be schooled explicitly. [on how to take care of users]

This utterance points to responsibility as a crucial factor in the construction of equity; however, there were differences in the distribution of responsibilities.

While the researchers generally highlighted their feelings of responsibility for the users and the project, the users' speech acts revealed another type of responsibility: the individual's responsibility to take care of him/herself.

If I accept to participate in something, then I contribute and do what the project requires or what I have accepted to do. And that's really my duty. And I let people know if I feel something was unfair or if I feel treated incorrectly. (User)

The above utterance not only reflects the users' responsibility vis‐à‐vis the project, it also underlines their moral right as human beings to be treated fairly.

Responsibility implies power and, thus, influences equity. The users were clear on the distribution of power. One user stated:

It is seldom the people in charge who notice the asymmetry; it is usually those beneath them.

The expression *those beneath them* suggests that the user might have assumed a subordinate position. The utterance also shows awareness of the unequal positions of users and the researchers and implies this inequity is less visible to researchers. However, another user stated that *the researcher needs to be trained to check with the user*.

This user thus assumed a position of responsibility with regard to his/her own interests.

The researchers drew exclusively on storylines involving responsibility for the research project and the users. In contrast, the users drew on storylines that implied responsibility for the project and for themselves.

### 4.3 Storylines of language and knowledge

Within these storylines, users and researchers showed huge engagement.

Some users felt that mastery of scientific language was essential for equity:

Researchers often express themselves very precisely, and if you don't understand, you might have trouble communicating, and this could lead to difficult relations. We do not speak the same language at all, so learning the language is absolutely important for being equal. (User)

This user constructs language as being tied to power; therefore, not ‘mastering’ the language might create a marginalized position. Although some users emphasized the importance of scientific language, they also protested the hierarchal positioning of language and the associated inequality:

#### We need to restrain the researchers and make sure they remain down to earth!

The colloquial expression ‘down to earth’ suggests the user considered the researchers' views too theoretical. Language and knowledge are concepts that are strongly tied to each other and to power. There was an on‐going negotiation between different types of language and knowledge. The researchers sometimes considered experiential and scientific knowledge equally important. At other times, however, they highlighted the value of their specialized knowledge.

I believe that anyone's knowledge is equally valuable. When a decision is to be made, I believe it's a matter of finding out who is most competent in that particular field. That is sometimes the user representative, and if I were the project leader, I would allow their opinions. We have different knowledge and competence, and this is important for the project. That's equity. (Researcher)

Another researcher stated:

I have the responsibility to facilitate and allow the user to come forth with his experiential knowledge.

Even if the researcher in the first excerpt considered both types of knowledge to be equal, the use of the term ‘allow’ in the excerpts suggests that the researchers might not have been convinced that both types of knowledge were equally valuable. The use of ‘allow’ also assumes a position in which the researcher has the right to decide who can ‘come forth’ with their knowledge.

The users emphasized the importance of their experiential knowledge. One user highlighted the fact that user knowledge was conveyed through everyday language:

We are not like researchers; we are individuals representing our daily lives.

Although the researchers stated that they considered both types of knowledge to be equivalent, some users expressed scepticism:

Some professionals believe their own competence is more valuable than our experiential knowledge.

One researcher offered the following rebuttal:

I believe the experiential knowledge that each person possesses is unique, and it is difficult for researchers to acquire it any other way.

Later in the interview, the same researcher mentioned the importance of methodology and scientific knowledge for conducting useful research. The following user utterance is a reminder of the two competing positions: *It's the professional versus the layman*. The foregoing statements suggest that researchers and users have different opinions regarding their own knowledge and that of others, which might also influence and challenge the position of equity in a research project.

### 4.4 Storylines of usefulness

Usefulness was the last topic portrayed as being central to equity.

According to one user:

Experiential knowledge becomes equal once one has identified where it is useful.

Another user stated, *I felt useful* [with my contributions], *and yeah, I felt like an equal part*.

A researcher pointed out the following:

The aspect of usefulness is important.

The feeling of having contributed towards a common goal, and thus having been valuable, seemed to be associated with a feeling of equity;

We have a common goal; we would like to reach it in the best possible way. (User)

The use of the pronoun ‘we’ supports this assumption, as it creates a sense of togetherness.

Usefulness, as articulated in the following utterance, was also related to efficiency through the notion of not wasting participants' time.

As a researcher, you request things that are useful for your project, but I think the Research Council has made some mistakes by requiring user involvement in all phases. It's not necessary for the users to be included in all phases. This might be a waste of time: both the researchers' and users' time. They should contribute with what they're really good at: their own experiences. (Researcher)

Although usefulness was discussed by both parties, the researchers tended to be more concerned with efficiency.

## 5 DISCUSSION

Our findings portray a picture that ‘equity’ was considered a crucial part of user involvement in research. Storylines about norms, responsibility, language, knowledge and usefulness evolved in the discussions and turned out to be central to both users and researchers. However, the analysis unveiled that the users' and researchers' storylines were not the same.

Existing literature underlines the importance of partnership and equity to ensure research quality where the ideal is to ‘share power’, rights and duties in the collaboration process.^14^, ^44^ Equity is a complex phenomenon, which is interpreted in many ways, and the claim that equity contributes to improved quality might be blurred by different understandings of equity. Within the storylines of norms and responsibility, the users' opinions were rooted in a universalist discourse that referenced the universality of human rights, which is in accordance with the findings of Gardinger,^11^ who identified that having an equal say in user involvement in research is considered a fundamental human right. In contrast, the researchers' opinions were mainly rooted in scientific discourse and prioritized the project itself through values such as taking responsibility for the research project. This finding is also in line with earlier findings that described a general trend of when groups with different power positions are positioned against each other; the less powerful focus on rights and the more powerful on duties,^45^ which explained why the users in the current study focused on rights while the researchers focused on duties.

Drawing on a universalist discourse facilitates decontextualization (moving from a research‐specific context to an everyday context), and it might explain the importance of human norms for the users. Through decontextualization, the higher status associated with the scientific context and the researchers' responsibilities (and accompanying power) were downplayed. Within the research community, users and researchers have different capacities for realizing their interests. Because the researchers assumed and were ascribed more responsibility (and thus, also rights) for the research project, they appeared more likely than the users to realize their interests. This finding is in line with the proposition that power is derived from the society of which an individual is a member.^46^

The storylines of knowledge and language were closely connected and related to status and power through discourses of lay and expert knowledge. While the traditional and dominant discourse is based on a process of knowledge transfer from experts to the public,^47^ within user involvement in health research, the value of both types of knowledge has been highlighted,^5^ even though equity seems difficult to achieve in practice. Scientific knowledge is considered to be generated from ‘objective’ measurements, while lay knowledge is based on the ‘subjective’ common sense of everyday life.^48^ Knowledge from different sources also entails different values, as highlighted in our study. Even though both parties acknowledged the importance of each other's knowledge, they generally drew on different storylines, implying unequal power and status. The data also indicated that the users, in particular, emphasized their own knowledge as being especially important and positioned experiential and scientific knowledge as equivalent. The negotiation of the values inherent in the users' knowledge might also have been seen as a way to gain access to the dominant discourse of science. When it comes to user involvement in research, lay knowledge is considered crucial to the development of scientific knowledge, and Bell and Pahl^13^ argue that the re‐positioning of knowledge is an important task that can provide new insights.

The force of speech acts, such as ‘make sure researchers remain down to earth’, might indicate user resistance to scientific knowledge. The attempt to draw researchers out of their social practice to gain equity might suggest a protest against the status and dominance of a discourse that privileges scientific language and knowledge. This aligns with earlier findings that state that although the lay public values expert knowledge, they increasingly wish to heed the importance of lay knowledge.^48^ It has been described as a paradox that political and institutional support for public participation does not problematize the notion of scientific expertise.^49^ It is likely that the resistance observed in our study may have been a sign of such a movement initiated by the lay representatives.

The picture is, however, not straightforward. Some user statements showed resistance towards the scientific discourse, while some suggested insecurity regarding the importance of scientific and lay knowledge. Some speech acts suggested that the parties acknowledged each other's knowledge, responsibilities and language, whereas others demonstrated resistance or disagreement. These findings confirm those of Davies and Harré,^50^ who recognized that individuals are not merely the bearers of knowledge produced by discourse but are capable of choosing among subject positions in different situations in relation to discursive practices. However, it seems that the researchers were mainly positioned within the scientific discourse. The users acknowledged that scientific discourse has more power, but at the same time, they positioned themselves so as to draw attention to marginalized discourses.

McClean and Shaw^51^ found that the common denominator for researchers and laypersons is the search for recognition of the ‘usefulness’ of their knowledge. This quest for recognition is reflected in the current study. While both parties mentioned usefulness, as also found in earlier studies,^52^ our analysis revealed different storylines. The users drew upon storylines related to providing new perspectives in the sense of adding value. In contrast, the researchers referenced storylines related to efficiency. These storylines were rooted in new public management discourse.^53^ However, being valuable and efficient were mentioned by the informants as being beneficial for the users and the researchers, respectively.

### 5.1 Methodological considerations

The use of heterogeneous focus groups is debatable, and interviewing each group separately might have yielded different data. It is likely that homogenous groups would have given the participants an opportunity to express potential inequity more explicitly; however, our purpose was to see how the participants constructed equity in a constellation similar to the involvement process in research. We recognize that it might be difficult for informants with different statuses to discuss equity due to their differing positions of power. However, power is an embodied phenomenon revolving within the positioning act, having the potential to influence others in a group.^20^ Most of the quotations were made by users and might have been related to a feeling of being safe in the focus group. The users were representing their organizations and were thus accustomed to voicing their opinions. Users without these skills and experiences might have responded differently.

## 6 CONCLUSION

We see it as interesting that initially, the analysis seemed to show a unified understanding of equity as both parties focused and described the same factors as important. Using positioning theory, we were able to identify that the apparent agreement masked that the users and researchers, through their storylines, were drawing on different discourses in the construction of equity: (a) general norms versus research‐specific norms, (b) individual responsibility versus responsibility to the project, (c) lay versus scientific knowledge and language and (d) efficiency versus feeling valuable. The storylines drew on two main discourses with different values and ideals related to different positions of power: a marginalized lay discourse and a dominant scientific discourse. Although the researchers and users drew upon both discourses, there was an emphasis on the scientific discourse, which privileged values such as status, responsibility and expertise. Because drawing on lay discourse might be seen as being in opposition to the dominant scientific discourse, through the use of alternative values in the construction of equity, the involvement process in research could be seen as a destabilization of academia as a privileged site for the production of knowledge.

User involvement in research is still a field ‘in the making’. It has been argued that research is active in the creation of reality and does not only access a pre‐existing reality.^13^, ^54^ Viewing the construction of equity through this assertion, one possible conclusion is that the negotiation of equity could contribute to the construction of new understandings and practices regarding equity in research processes. Our study revealed different perceptions about ‘equity in user involvement’ and implies that it is important to uncover and discuss these differences in collaboration processes.

## CONFLICT OF INTEREST

The authors declare that there is no conflict of interest.

## AUTHOR CONTRIBUTIONS

All authors contributed in conducting this study and writing the article. SS and ICF conducted the interviews and SS transcribed the interviews. SS, ICF and IRK read the interviews and conducted the analysis. All authors read and approved the final manuscript.

## ETHICAL APPROVAL

The project fell outside the jurisdiction of the Medical and Health Research Ethics Committee; however, because the interviews were audio‐recorded, approval was requested and received from the Norwegian Centre for Research Data.

## Data Availability

The data that support the findings of this study are available from the corresponding author [SS] upon reasonable request.
